# Some Abdominal Cases*Read before Richmond Bcademy of Medicine and Surgey, June 12, 1898.

**Published:** 1898-08

**Authors:** George Ben Johnston

**Affiliations:** Richmond, Va.; Professor of Gynecology and Abdominal Surgery, Medical College of Virginia; Fellow of the American Surgical Association; Member of the Southern Surgical and Gynecological Association, etc.; 407 East Grace Street


### SOME ABDOMINAL CASES.*

BY GEORGE BEN JOHNSTON, M. D., RICHMOND, VA.

Professor of Gynecology and Abdominal Surgery, Medical College of Virginia; Fellow of the American Surgical Association; Member of the Southern Surgical and Gynecological Association, etc.

It is a source of regret to me that I have not for the past ten or fifteen years kept the pathologic specimens that have come from the various operations I have performed, because great benefit may be derived later on from a correct study of these specimens. With this in view, for the last few months I have kept all specimens worth preserving, concerning a few of which I speak to you to-night, giving a brief history of the cases from which they were obtained.

CASE I.—The first specimen I wish to show was taken from Mrs. R. D. B., aged twenty-four years, referred to me by Dr. J. P. Haller, Pocahontas, Va. Admitted to the Old Dominion Hospital January 17, 1898. Previous history uneventful save an attack of typhoid fever six years ago. Married six years; one child five years of age; no miscarriages. For two years has suffered with leucorrhea and very painful menstruation. Periods have not appeared for five months.

An examination of this case, made on the 18th day of January, revealed a large, globular tumor in the lower part of the abdomen, some enlargment and swelling of the breasts, discoloration of the nipples, and fluid in the breasts. On minuter examination fetal movements could be feebly discerned. A digital examination by the vagina revealed a large and eroded cervix, and inspection discovered a large ulcer upon the cervix, which turned out to be a carcinoma.

This condition confronted us: Here is a woman only twenty-four years of age presenting a carcinoma of the cervix, complicated by pregnancy advanced to the fifth month. Complete hysterectomy was the only course for her relief. I thought it a pity to sacrifice the child, and therefore determined to keep her under observation to discover whether or not the disease was making rapid strides. It was ascertained that the spread was slow. I therefore determined it would be justifiable, so far as the woman was concerned, to defer any operative interference until the child was at least viable. I recommended a return to the hospital after the termination of the seventh month of pregnancy.

She was readmitted to the hospital on the 13th day of March and was operated upon on the 22d. The operation was the Porro, and the anesthetic used was chloroform. All of the ordinary preparations of the patient were made. The first step in the operation was the complete destruction of the cancerous tissue of the cervix by means of the galvano cautery.

*Read before Richmond Bcademy of Medicine and Surgey, June 12, 1898.

A long, free incision was made in the abdominal wall, and quickly carried down to the uterus which was exposed and delivered through the wound. Two loops of a large elastic ligature were thrown around the cervix but not tightened. An assistant grasped the cervix firmly with the hand to control bleeding. The uterus itself was then incised by a free and rapid incision and immediately the child was delivered, cord clamped, and child turned over to the accoucher. The ligature around the cervix was at once tightened and clamped, and when this was completed a large pad of gauze was put into the uterus and two stitches of pedicle silk were made to close the uterine incision. No attempt was made to dislodge the placenta, and a gauze sponge was placed in for the purpose of absorbing any oozing that might occur, thus diminishing the risk of contaminating the peritoneal cavity. In the meantime the intestines had been protected by large sheets of gauze. From this point on, the operation was one of simple hysterectomy. The ovarian vessels were ligated, divided between the ligatures and then the uterine arteries were secured, the vagina opened from above, the curvix dissected out, as was also the upper portion of the vagina. As soon as this was accomplished, the proper toilet of the peritoneum was made and the wound closed by through-and-through silk-worm gut sutures. I have long abandoned the practice of closing the abdominal wound with tyers of sutures, using only through-and-through sutures of silk-worm gut. Vaginal drainage was used.

This woman made a very happy recovery. The child, which was very poorly nourished, perished at the end of two and a half hours. The second day there appeared in the breasts a considerable flow of milk which was suppressed by the application of the so-called Murphy jacket. The specimen from this case shows the uterus with the fetal membranes. I was much struck in this instance, as I have been in others, to see the extent to which very muscular specimens shrink after removal.

CASE. II.—The next specimen, a rare one as presenting a very curious combination of pathologic conditions, is from the case of Mrs. A. V. W., referred to me by Dr. H. C. Beckett, Clover, Va. This patient, a white female, aged fifty years, gave the following history: Married at twenty-seven, but never had any children. Menstruated at seventeen, after which courses were regular, but attended with much pain since marriage. Has considerable leucorrhea and bloody discharge. About eighteen years ago consulted a physician in this city, who told her she had a small fibroid which would dissappear at change of life. Not troubled again until June of last year, since which time she has had six attacks, each of increasing severity, of intense pain in abdomen, accompanied by some bloody discharge, and confining her to bed for several days during each attack. Admitted to the Old Dominion Hospital May 3, 1898.

An examination of this patient revealed the uterus perhaps five times as large as normal, nodular, and somewhat depressed, the cervix being within easy reach of the examining finger, which also revealed an ulcer upon the cervix and the further fact that carcinoma of the cervix was complicated by fibroids of the uterus.

A complete hysterectomy was undertaken and was free from incident. Upon removal of the uterus it was discovered that not only were there fibroids in the structure of this organ, but also a subserous fibroid, situated on the posterior surface of the uterus and about the size of a small walnut, which had undergone *calcareous degeneration* here shown. You can hear the sound as it is struck by the nail. It was covered only by peritoneum with an absence of all other tissue. In the body of the uterus other masses were plainly seen. This patient also made a perfectly satisfactory recovery and has returned home.

CASE III.—One of the most interesting specimens I have to show you is that taken from a negro woman, Maria Prosser, referred to me by Dr. C. M. Miller of this city. This patient, aged forty-eight years, was married at nineteen, and has had fifteen children, triplets once. Menopause six or seven years ago. No previous illness. Complains of severe pain and enlarged abdomen, which symptoms were first noticed about four years ago. Pain intermittent. Four months ago had very severe attack of pain and rapid increase of swelling. Had general edema and some congestion of kidneys, due to pressure. Occasional discharge from vagina, but no hemorrhage.

When I first saw her, in consultation with Dr. Miller, she was much debilitated, greatly reduced, thoroughly anemic, abdomen enormously swollen, not only from the tumor but also from a collection of ascitic fluid, and edema of the lower extremities. Examination showed a large, irregular tumor occupying lower portion of the abdomen, extending above the umbilicus, higher on the right than on the left side. The abdominal walls were very thin. Prominently shown above the symphigis was a protuberance half the size of a cocoanut, movable from side to side and plainly attached to the tumor. The neck of the uterus was out of reach of the examining finger. The mass resembled a full bladder displaced by a pelvic tumor, but was pronounced to be the uterus with tumor attached. The diagnosis made was intraligamentous cyst of right side with the uterus lifted out of the pelvis. Deeming it unwise to operate in her present condition, she was put under a preparatory course and then operated upon at the Old Dominion Hospital on May 21st.

After the incision was made and the tumor exposed, the accuracy of the diagnosis was verified. Ordinarily this would have been an extremely difficult case and, in all likelihood, radical operative interference would have proved fatal. Goodell says, "These are the patients that die on the table."

We are indebted to Dr. Rufus B. Hall,* of Cincinnati, for a recent method of dealing with intraligamentous cysts which renders the operation almost as safe as an ovariotomy. The old method was to split the periteneum, then proceed to enucleate the cyst. As the blood supply is very large this meant very profound and sometimes fatal hemorrhage. The operator was embarrassed by the great flow of blood, had to work with the utmost rapidity, his manipulations had to be carried on by the sense of touch and not by sight. Having heard Dr. Hall describe his method at the recent meeting of the Southern Surgical and Gynecological Association, where he exhibited a specimen very similiar to the one before us, I concluded to try his method in this case, and found it to work admiraby.

I proceed to do a supra-vaginal hysterectomy and was able to control the blood supply to the tumor. I ligated the ovarian artery on the healthy side with a double ligature, then severed the tissues between the ligatures, went down to the uterine artery on the same side and ligated it, after securing which I returned to the affected side and ligated the ovarian artery. This left no vessel of magnitude, save the uterine artery on the affected side. I then severed the cervix from the good to the affected side until I reached the uterine artery, passed a ligature around this and proceed to lift the tumor out. *Practically no blood was lost.*

After removal the tumor was discovered to have three very large cavities filled with fluid and accumulated blood. One of these cavities contained a clot very finely organized; in the others the fluid was thin and left clean walls.

This woman did extremely well for the first three days; then, in the temporary absence of the nurse, got up and sat in a chair. Untoward symptoms developed, and her life was dispaired of, but the symptoms subsided and an excellent recovery followed, the patient leaving the hospital at the end of six weeks. This case is of particular interest, as occurring in a negro woman. Tumors of this type in the negro race are even rarer than ovarian tumors, which latter are almost never seen. This specimen may, therefore, be regarded as a surgical curiosity.

The next specimen is that taken from a woman recently admitted to the Old Dominion Hospital, referred to me by Dr. R. W. Fry, of Roanoke. Mrs. M. C. W., aged thirty-five years; married. Previous history: Menstruated at fifteen; regular ever since. Married at eighteen; three children and five miscarriages; last three due to lacerated cervix. Typhoid fever at the age of twenty-five. Says she has suffered with congestion of the womb for ten years, and has been treated

*Trans. South. Surg. and Gyn. Assn., Vol. X, p. 184.

for ulceration of the womb. Pain is most severe between periods; is located in lower part of the abdomen, extending down the thighs, especially the right one. Dr. Fry examined patient, and, finding a mass in the pelvis to right of uterus, diagnosed some form of ovarian tumor and referred the case to me. Admitted to the Old Dominion Hospital June 8, 1898. On the right side I found a mass the size of a large orange; on the opposite side there was an enlarged fallopian tube, the nature of which I was unable to make out, though convinced it was a hydrosalpinx of pyosalpinx. The ovary on the left side was enlarged and very firm.

Operated upon on June 11th. Upon opening the abdomen it was found that the entire pelvis was domed over by a mass of matted adhesions, the like of which I have rarely seen. It seemed impossible to enter this roof in order to get down into the crevices where lines of cleavage could be established. After a time we were successful in our attempt and began to excavate the uterus and its appendages. When this was accomplished the left tube was found in a state of hydrosalpinx as large as the thumb of a good-sized hand. The ovary on the same side contained a hematoma. The mass on the right side was discovered to be an ovarian abscess. Supra-vaginal hysterectomy was decided upon and accomplished in the usual way, after which a glass drain was inserted. The patient made an excellent recovery. This is a very interesting specimen as showing a multitude of pathologic conditions.

CASE V.—Here is another specimen, which I regret to say has been practically destroyed by the evaporation of the alcohol from the preserving fluid. It comes from a woman referred to me by Dr. J. Bolling Jones, of Petersburg. Miss A. L. F., white, aged thirty-eight years, was in good health up to sixteen years ago. At that time noticed a protrusion of the cervix. This has given her much discomfort. Marked nervousness; indigestion and nausea. Admitted to the Old Dominion Hospital December 7, 1897.

It was perfectly easy to diagnose hypertrophied cervix, descended uterus, fibroids (which were everywhere to be felt over the fundus of the uterus), and also a tumor the size of a cocoanut to the right of the uterus, but as to what this tumor was, I felt uncertain. I gave the diagnosis of probable dermoid cyst and the operation proved its correctness.

The uterus was found to contain a number of fibroids varying in size from a hickory nut to a small lemon. Apparently all of them were subserous. It was therefore not necessary to remove the uterus, but was to do myomectomies. Therefore these four fibroids were enucleated and the button-holes through which they were removed from under the peritoneum were stitched up by Dembert sutures. The mass to the right of the uterus was enucleated, raised out of the pelvis and removed by the ordinary method of ligating its pedicle. A ventro fixation was next performed. The cervix was found hypertrophied and within the vagina. As a matter of safety the undiseased ovary was removed, hoping to procure an atrophy of the uterus, diminishing the elongated cervix and rendering the fixation more safe. These objects were accomplished by the operation.

On examining the tumor it was found to be a dermoid cyst, filled with the characteristic cheesy masses and oily fluid, and contained a great deal of long, coarse, brown hair growing very abundantly from the cyst wall. Teeth, bone, serous and mucous membranes are occasionally found in these tumors, and by one or two observers it has been reported that tissue resembling brain substance has been found in them. This patient made a first-rate recovery.

407 East Grace Street.

